# Antibody deficiency in myotonic dystrophy type 1: A differential diagnosis below the radar

**DOI:** 10.70962/jhi.20250223

**Published:** 2026-09-30

**Authors:** Edoardo Galli, Christoph T. Berger, Thomas Daikeler, Anne-Kathrin Peyer Kauffmann, Marc Emmenegger, Armin Droll, Andrea Egger, Michael Sinnreich, Mike Recher

**Affiliations:** 1 Department of Neurology, University and University Hospital of Basel, Basel, Switzerland; 2 Department of Neurology and Neurophysiology, University Hospital Freiburg, Freiburg, Germany; 3 University Center for Immunology, University Hospital Basel, Basel, Switzerland; 4 Division of Medical Immunology, Department of Laboratory Medicine, University Hospital Basel, Basel, Switzerland; 5 Department of Internal Medicine, Regional Hospital Dornach, Dornach, Switzerland; 6 Endocrinology Private Practice, Basel, Switzerland; 7 Department of Biomedicine, Neuromuscular Research Group, University of Basel, Basel, Switzerland; 8 Immunodeficiency Laboratory, Department of Biomedicine, University of Basel, Basel, Switzerland

## Abstract

Myotonic dystrophy type 1 is a rare, underdiagnosed genetic neuromuscular disorder that is often accompanied by hypogammaglobulinemia, serving as a crucial diagnostic clue. We report the clinical and immunophenotypic features of two patients and discuss the underlying pathogenesis.

## Teaser

Galli, Recher et al. describe two patients with hypogammaglobulinemia associated with myotonic dystrophy type 1 (DM1), a rare and underdiagnosed monogenic neuromuscular disorder. The authors present clinical and immunophenotypic clues to aid clinical immunologists in making a correct diagnosis, while discussing the knowns and unknowns in the molecular pathogenesis of DM1-related antibody deficiency.

## Introduction

Myotonic dystrophy type 1 (DM1, OMIM# 160900, ORPHA:273) was first described in 1901 and has an estimated prevalence of 1:10,000, with geographical hotspots (1). The disease is underdiagnosed, especially when neuromuscular features are subtle. Besides the eponymous neuromuscular features, DM1 is a multisystem disease affecting various organs, including the central nervous system, the heart, the eyes, the gastrointestinal system, and endocrine organs (1). DM1 results from CTG nucleotide expansions in the 3′ noncoding mRNA of the myotonic dystrophy protein kinase (*DMPK*) gene. These abnormal transcripts sequester RNA-binding proteins and alter RNA splicing of developmentally regulated genes, explaining the multisystem involvement in DM1.

Hypogammaglobulinemia in DM1 was first documented in the literature in 1956 and was associated with decreased in vivo half-life of IgG, but not IgM or IgA, already in 1966 (2). Recent cohort studies reveal hypogammaglobulinemia in ∼40% of DM1 patients (3).

*DMPK* is currently not recognized as an official inborn error of immunity (IEI) gene as defined by the International Union of Immunologic Societies (4). This fact may reinforce a lack of awareness of this differential diagnosis in the evaluation of antibody-deficient patients.

Here, we describe the clinical, serologic, and immunophenotypic features of two patients with DM1.

## Results

Patient 1 was a Caucasian male referred to the immunology clinic at the age of 47 years for suspected retinal vasculitis. The patient’s vision had not improved after cataract surgery. He had been diagnosed with DM1 at the age of 40 years following surgery for lower back pain (discus hernia), when inappropriate muscle weakness was noticed. Creatine kinase (CK) serum levels were mildly elevated (Table 1). Radiologic imaging and immunologic evaluation, including testing for anti-neutrophil cytoplasmic antibodies, revealed no evidence of systemic or cerebral vasculitis. A substantial reduction of serum IgG to 4.4 g/L (reference range 7–16 g/L) was noted with a reduction of all IgG subclasses (Table 1). IgM was within the reference range. Lymphocyte subpopulations were unremarkable, as were B cell subpopulations (Table 1). Due to a vasculitis-like pattern in the retinal fluorescein angiography, an initial therapy with systemic steroids and a basic immunomodulation with methotrexate was started with reported improvement in vision. As the patient at the time of immunologic evaluation did not report recurrent infections, immunoglobulin replacement therapy (IgRT) was not initiated at that time, and a vaccination update was recommended. The patient was then lost to follow-up at our institution.

**Table 1.** CK activity, albumin concentration, and immunophenotypic analysis in two patients diagnosed with DM1

Laboratory value	Reference range	Patient 1	Patient 2
**Clinical chemistry**	​	​	​
CK (U/L)	50–200	**360**	**448**
Serum albumin (g/L)	35–52	38	42
**Immunoglobulins**	​	​	​
IgG (g/L)	7.0–16.0	*4.4*	*4.1*
IgG1 (g/L)	4.90–11.40	*3.11*	*2.22*
IgG2 (g/L)	1.50–6.40	*1.03*	*1.35*
IgG3 (g/L)	0.20–1.10	*0.13*	*0.12*
IgG4 (g/L)	0.08–1.40	*0.05*	0.08
IgA (g/L)	0.70–4.00	1.57	0.79
IgM (g/L)	0.40–2.30	0.74	0.84
IgE (kIU/ml)	<100	N.D.	<5
**Lymphocyte subpopulations**	​	​	​
T cells relative (% of lymphocytes)	55–86%	81%	**88%**
T cells absolute (/μl)	742–2,750	1,624	1,933
B cells relative (% of lymphocytes)	5–22%	8%	8%
B cells absolute (/μl)	80–616	171	168
NK cells relative (% of lymphocytes)	5–26%	10%	*4%*
NK cells absolute (/μl)	84–724	211	96
CD4^+^ T cells relative (% of lymphocytes)	33–58%	**65%**	**67%**
CD4^+^ T cells absolute (/μl)	404–1,612	1,259	1,479
CD8^+^ T cells relative (% of lymphocytes)	13–39%	15%	20%
CD8^+^ T cells absolute (/μl)	220–1,129	290	446
**B cell subpopulations**	​	​	​
Naïve B cells relative (% of B cells)	25.1–92.4%	59.6%	35.4%
Naïve B cells absolute (/μl)	66–228	102	59
Marginal zone–like B cells relative (% of B cells)	3.1–59.7%	14.1%	36.5%
Marginal zone–like B cells absolute (/μl)	8–172	24	61
Class-switched memory B cells relative (% of B cells)	2.4–32.6%	21.5%	23.1%
Class-switched memory B cells absolute (/μl)	8–102	37	39
CD21^lo^ B cells relative (% of B cells)	0.5–4.7%	3.2%	3.0%
CD21^lo^ B cells absolute (/μl)	1–12	5	5
Transitional B cells relative (% of B cells)	0.3–2.9%	2.6%	0.6%
Transitional B cells absolute (/μl)	1–5	4	1
Plasmablasts relative (% of B cells)	0.1–3.0%	0.4%	1.4%
Plasmablasts absolute (/μl)	1–5	1	2
**CD4** ^+^ **T cell subpopulations**	​	​	​
CD4^+^ naïve relative (% of CD4^+^ T cells)	15.7–54.7%	N.D.	23.6%
CD4^+^ central memory (% of CD4^+^ T cells)	8.0–28.9%	N.D.	10.6%
CD4^+^ effector memory (% of CD4^+^ T cells)	16.8–57.4%	N.D.	55.3%
CD4^+^ TEMRA (% of CD4^+^ T cells)	3.6–23.2%	N.D.	10.4%
CD4^+^ recent thymic emigrants (% of CD4^+^ T cells)	14.1–37.2%	N.D.	18.4%
CD4^+^ follicular T helper (% of CD4^+^ T cells)	6.9–19.1%	N.D.	11.1%
CD4^+^ regulatory T cells (% of CD4^+^ T cells)	6.1–11.0%	N.D.	6.5%
CD4^+^ activated (% of CD4^+^ T cells)	4.1–15.6%	N.D.	8.3%
**CD8** ^+^ **T cell subpopulations**	​	​	​
CD8^+^ naïve relative (% of CD8^+^ T cells)	7.0–62.5%	N.D.	*6.2%*
CD8^+^ central memory (% of CD8^+^ T cells)	0.6–4.4%	N.D.	*0.5%*
CD8^+^ effector memory (% of CD8^+^ T cells)	4.3–64.5%	N.D.	25.5%
CD8^+^ TEMRA (% of CD8^+^ T cells)	8.1–60.5%	N.D.	**67.8%**
CD8^+^ activated (% of CD8^+^ T cells)	8.7–45.2%	N.D.	31.9%
**TCR-** **α** **/** **β** **; TCR-** **γ** **/** **δ**	​	​	​
TCRab (% of lymphocytes)	36.0–98.0%	N.D.	85.4%
TCRgd (% of lymphocytes)	0.8–11.0%	N.D.	0.8%
**Pneumococcus serotype–specific IgG**	**Reference range**	**Patient 2 before vaccination**	**Patient 2 after vaccination**
IgG (g/L)	7.0–16.0	*4.1*	*3.3*
Anti-pneumococcus (4) IgG (mg/L)	>0.3	*<0.3*	*<0.3*
Anti-pneumococcus (6B) IgG (mg/L)	>0.3	*<0.3*	2.2
Anti-pneumococcus (9V) IgG (mg/L)	>0.3	0.3	0.9
Anti-pneumococcus (14) IgG (mg/L)	>0.3	<0.3	1.9
Anti-pneumococcus (18C) IgG (mg/L)	>0.3	<0.3	0.4
Anti-pneumococcus (19F) IgG (mg/L)	>0.3	1.4	2.9
Anti-pneumococcus (23F) IgG (mg/L)	>0.3	5.0	>5.0
Anti-pneumococcus (8) IgG (mg/L)	>0.3	*<0.3*	>5.0
Anti-pneumococcus (9N) IgG (mg/L)	>0.3	1.1	1.9
Anti-pneumococcus (10A) IgG (mg/L)	>0.3	*<0.3*	0.6
Anti-pneumococcus (11A) IgG (mg/L)	>0.3	*<0.3*	>5.0
Anti-pneumococcus (15B) IgG (mg/L)	>0.3	0.4	>5.0
Anti-pneumococcus (17F) IgG (mg/L)	>0.3	*<0.3*	1.5
Anti-pneumococcus (20) IgG (mg/L)	>0.3	0.4	2.8

In patient 2, pre- and postvaccination (PCV20 vaccine, Prevenar 20) IgG against the indicated pneumococcus serotypes were determined in addition. Values above the reference range are indicated in bold, and values below the reference range are marked in italics. TEMRA, terminal effector cells reexpressing CD45RA.

Patient 2, a 45-year-old Caucasian male, was referred to our university immunology center for suspected common variable immunodeficiency (CVID) after hypogammaglobulinemia was detected. Immunoglobulins had been measured after two urinary tract infections within 1 mo. The medical history revealed a diagnosis of pseudohypoaldosteronism type 2 in 2021, when high serum potassium levels in the absence of renal failure were noted. In 2023, dermatologic evaluation revealed a pilomatricoma, which was surgically removed.

Immunologic evaluation showed a reduced serum IgG of 4.1 g/L (3.3 g/L at repeat testing), while IgM and IgA concentrations were within the reference range. Consequently, IgG subclasses were reduced (Table 1). Serum IgE was below the detection limit. Serotype-specific IgG against pneumococcus was measured before and after vaccination with the 20-valent conjugate vaccine (PCV20, Prevenar-20). After vaccination, patient 2 mounted protective IgG quantities despite persisting total serum IgG reduction (Table 1). As in patient 1, B cell phenotyping showed unremarkable B cell subpopulation proportions and absolute cell numbers (Table 1). The T cell compartment was overall unremarkable (Table 1). Besides an evident facies myotonica, percussion of the thenar eminence produced a myotonic response (percussion myotonia), which was also evident after forced handgrip bilaterally (handgrip myotonia). The clinical and laboratory features raised suspicion of DM1.

Genetic testing for CTG repeats in the 3′UTR of the *DMPK* gene showed a heterozygous triplet expansion of >150 repeats (normal <37). Further assessments for associated systemic complications were organized with our neuromuscular center.

## Discussion

DM1 is an important differential diagnosis in patients with hypogammaglobulinemia. As in patient 2, antibody deficiency may be an objective and important clue leading to diagnosis of DM1. CVID was unlikely in both patients, given the isolated reduction in serum IgG, with IgA and IgM within the reference range. In addition, B cell subpopulations did not show CVID-typical changes, e.g., reduced memory B cells. Pneumococcus vaccination induced protective serotype-specific IgG levels in patient 2, indicating robust B cell function.

While hypogammaglobulinemia is a risk factor for recurrent bacterial infections, it is unclear at which point IgRT should be initiated in DM1 due to the lack of prospective studies. The urinary tract infections reported in patient 2 are not a typical consequence of antibody deficiency. We therefore decided against immunoglobulin substitution in that patient.

The molecular mechanism underlying hypogammaglobulinemia in DM1 remains controversial. The DM1-associated reduced IgG half-life suggests dysfunction of the neonatal Fc receptor (FcRn). Indeed, the gene encoding FcRn, *FCGRT*, is located on the same chromosome as *DMPK* (chromosome 19) and long-range gene inactivation in cis by mutated *DMPK* mRNA has been postulated.

The clinical relevance of impaired FcRn-dependent IgG recycling is illustrated by familial hypercatabolic hypoproteinemia (FHH), a rare disease caused by mutations in the *B2M* gene encoding β2-microglobulin, which is required for stable FcRn expression and function. FHH is associated with low IgG and low albumin concentrations and provides proof of principle that defective FcRn biology may accelerate IgG catabolism. No genetically determined disease caused by mutations in *FCGRT* has been described to date. While FHH causes low serum IgG plus low serum albumin concentrations, albumin was unremarkable in both patients presented here (Table 1) and is commonly within the expected range in DM1. Thus, a simple reduction of FcRn expression likely does not explain the immunoglobulin and albumin findings in DM1 patients. Notably, not all FcRn inhibitors, a novel class of immune modulators used for various autoimmune diseases such as myasthenia gravis, reduce serum albumin in addition to lowering serum IgG (5). Thus, the lack of albumin reduction in DM1 does not preclude FcRn as the molecular target in DM1-dependent hypogammaglobulinemia. Although the existence and physiological relevance of *FCGRT* splicing variants in humans remain controversial, it may be speculated that *DMPK* repeat–mediated splicing dysregulation could skew *FCGRT* transcripts toward nonfunctional isoforms that are crucial in antibody recycling process but do not participate in albumin homeostasis. While the aim of the current letter is to put DM1 on the map in the differential diagnosis of hypogammaglobulinemia, we also encourage the IEI community to further elucidate the underlying molecular pathogenesis.

The personal history of DM1 patients may include nonimmunologic clues that help to make the correct diagnosis. Ophthalmologic complications such as ptosis, corneal dystrophy, early-onset cataracts, low intraocular pressure, and retinal pigment anomalies are well described in DM1, while recent observations with novel methodology, like optical coherence tomography angiography, find common retinal vascular anomalies. Thus, the observed retinal vascular changes in patient 1 may have represented a prominent form of DM1-associated retinal vasculopathy.

The pilomatricoma in patient 2 is typically associated with DM1, although these benign skin tumors of hair follicle origin can also be found in other IEI such as Kabuki syndrome. While rarely mentioned in the DM1-related literature, pseudohypoaldosteronism has also been reported.

In summary, immunologists may encounter undiagnosed DM1 patients in the evaluation of hypogammaglobulinemia. A combination of isolated IgG reduction with unremarkable serum IgM and IgA and mild CK elevation should prioritize the differential diagnosis of DM1, if no plausible alternative explanation is found. Adequate vaccination responses and unremarkable B cell subsets may support DM1 diagnosis. The personal history may, as in the cases presented here, reveal common or rare features of DM1 multisystem involvement. After a clinical and molecular diagnosis of DM1, interdisciplinary workup involving the primary care physician is mandatory for optimal treatment. Diagnostic delay may cause life-threatening complications such as unnoticed cardiac involvement. IgRT might be required in case of very low IgG (e.g., <4 g/L) plus recurrent infections and may require higher dosage due to the reduced IgG half-life. We propose to reevaluate *DMPK* as an official IEI gene to augment the visibility of this entity for immunologists and IEI specialists.

## Data Availability

All data are either included in the manuscript or available upon request.
